# Integrating MRI-based tumour staging within the TNM classification system in modern prostate cancer management

**DOI:** 10.1186/s13244-026-02346-w

**Published:** 2026-07-09

**Authors:** Georgios Agrotis, Andrei Purysko, Anwar Padhani, Fredrik Jäderling, Geert Villeirs, Samuel J. Withey, Maarten de Rooij, Tristan Barrett, Ivo G. Schoots

**Affiliations:** 1 Department of Radiology, The Netherlands Cancer Institute, Amsterdam, The Netherlands; 2 GROW School for Oncology and Reproduction, Maastricht University, Maastricht, The Netherlands; 3 Section of Abdominal Imaging and Nuclear Radiology Department, Imaging Institute, Cleveland Clinic, Cleveland, OH USA; 4 Paul Strickland Scanner Centre, Mount Vernon Cancer Centre, London, United Kingdom; 5 Department of Radiology, Capio St Göran’s Hospital, Stockholm, Sweden; 6 Institution of Molecular Medicine and Surgery (MMK), Karolinska Institute, Stockholm, Sweden; 7 Department of Radiology, Ghent University Hospital, Ghent, Belgium; 8 Department of Radiology, Royal Marsden Hospital, London, United Kingdom; 9 Department of Medical Imaging, Radboud University Medical Center, Nijmegen, The Netherlands; 10 Department of Radiology, Cambridge Biomedical Campus, Addenbrooke’s Hospital & University of Cambridge, Cambridge, United Kingdom; 11 Department of Radiology and Nuclear Medicine, Erasmus University Medical Center, Rotterdam, The Netherlands

**Keywords:** Prostate cancer, Magnetic resonance imaging, Digital rectal examination, Extraprostatic extension, Tumour staging

## Abstract

**Abstract:**

MRI has transformed prostate cancer diagnosis, risk stratification, and treatment planning. However, the tumour-node-metastasis (TNM) classification continues to rely exclusively on digital rectal examination (DRE) for clinical T-staging, despite DRE’s limited diagnostic accuracy and prognostic performance. This mismatch between modern MRI practice and outdated staging criteria undermines prognostication and treatment decision-making. In this review, we synthesise current evidence on DRE- and MRI-based local staging, discuss the limitations of the current TNM framework, and present a proposal for parallel reporting of MRI-based T-staging (mrT) alongside clinical T-staging (cT). We further outline the rationale for developing MRI-derived prognostic groups, benchmarked against pathology and long-term oncologic outcomes, and present a framework intended to support future evidence-based revisions of the official TNM classification.

**Critical relevance statement:**

Clinical adoption of prostate MRI, TNM T-staging still relies on digital rectal examination, a method with limited sensitivity and prognostic value. This discordance leads to stage misclassification and suboptimal risk stratification. Parallel reporting of MRI-based T-staging and development of MRI-derived prognostic groups are essential to align staging with contemporary imaging practice, support evidence-based treatment decisions, and inform future revisions of the TNM classification system.

**Key Points:**

- Digital rectal examination (DRE) is inferior to MRI for detecting extraprostatic extension.
- MRI-based and DRE-based T-staging should be reported separately to evaluate stage migration, as MRI-based T-staging may shift patients into higher stages without reflecting true biological risk.
- Parallel reporting of MRI-based T-staging and development of MRI-derived prognostic groups are essential to align staging with contemporary imaging practice, support evidence-based treatment decisions, and inform future revisions of the TNM classification system.

**Graphical Abstract:**

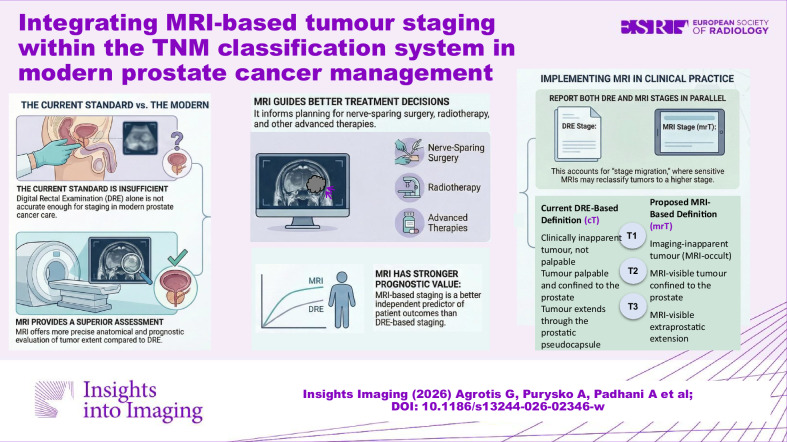

## Introduction

Accurate tumour (T) staging remains a cornerstone of prostate cancer management, treatment selection, and guiding prognostic outcomes [1, 2]. Despite significant advances in imaging, the 9th edition of the Union for International Cancer Control (UICC) Tumour Node and Metastasis (TNM) classification still bases T-staging exclusively on clinical digital rectal examination (DRE) (Table 1a) [3]. The reliance on clinical palpation as the defining criterion of tumour extent reflects historical practice rather than contemporary capabilities.

**Table 1:** TNM classifications

(a) UICC 9th edition TNM classification		(b) Proposed MRI-based TNM classification	
**cT—primary tumour**		**mrT—primary tumour**	
**cTX**	Primary tumour cannot be assessed	**mrTX**	Primary tumour cannot be assessed
**cT0**	No evidence of primary tumour	**mrT0**	No evidence of primary tumour
**cT1**	**Clinically inapparent tumour, not palpable**	**mrT1**	**Imaging-inapparent tumour (MRI-occult)**
	**cT1a**—Incidental histologic finding in ≤ 5% of tissue resected		-
	**cT1b**—Incidental histologic finding in > 5% of tissue resected		-
	**cT1c**—Tumour identified by needle biopsy (usually elevated PSA)		-
**cT2**	**Tumour palpable and confined within the prostate**	**mrT2**	**MRI-visible tumour confined within the prostate**
	**cT2a**—Involves ≤ half of one lobe	**mrT2a**	Involves ≤ half of one lobe
	**cT2b**—Involves > half of one lobe, not both	**mrT2b**	Involves > half of one lobe, not both
	**cT2c**—Involves both lobes	**mrT2c**	Involves both lobes
**cT3**	**Tumour extends through prostate capsule**	**mrT3**	**MRI-visible extraprostatic extension**
	**cT3a**—Extraprostatic extension (incl. microscopic bladder-neck invasion)		**mrT3a**—Extraprostatic extension
	**cT3b**—Seminal vesicle invasion		**mrT3b**—Seminal vesicle invasion
**cT4**	**Tumour fixed or invading adjacent structures**	**mrT4**	**MRI-visible invasion of adjacent structures other than seminal vesicles**
**cN—Regional lymph nodes**		**mrN—Regional lymph nodes**	
**cNX**	Nodes cannot be assessed	**mrNX**	Nodes cannot be assessed
**cN0**	No regional lymph node metastasis	**mrN0**	No regional lymph node metastasis
**cN1**	Regional lymph node metastasis	**mrN1**	Regional lymph node metastasis
**cM—Distant metastasis**		**mrM—Distant metastasis**	
**cM0**	No distant metastasis	**mrM0**	No distant metastasis
**cM1**	**Distant metastasis**	**mrM1**	**Distant metastasis**
	**M1a**—Non-regional lymph nodes		**M1a**—Non-regional lymph nodes
	**M1b**—Bone metastasis		**M1b**—Bone metastasis
	**M1c**—Other sites		**M1c**—Other sites

Meanwhile, magnetic resonance imaging (MRI) has evolved into the diagnosis, treatment, and prognosis of prostate cancer worldwide, guiding targeted biopsies, tailoring surgical and radiotherapy planning, and supporting active surveillance decisions. This divergence between what clinicians use in practice (MRI) and what the TNM staging system enforces (DRE) has created a structural misalignment within modern prostate cancer management. Consequently, crucial MRI-derived information is currently excluded from the TNM T-stage, creating misalignment that is detrimental to risk stratification and clinical decision-making.

This review aims to summarise the limitations of DRE-based clinical T-staging and the contemporary role of MRI in local prostate cancer staging, to discuss why parallel reporting of cT and mrT is currently preferable to direct substitution within existing staging systems, and to outline a framework for future MRI-calibrated staging and prognostic grouping.

## Disconnecting modern practice from clinical T-staging

Current prostate cancer management relies on guideline-based risk stratification rather than anatomical T-stage alone. In widely used systems such as the EAU, NCCN, and D’Amico classifications, clinical T-stage derived from DRE is already incorporated alongside PSA and biopsy Grade Group/ISUP score to guide treatment selection, including active surveillance, local treatment, or treatment intensification. The AJCC prognostic stage grouping similarly combines anatomical tumour extent with PSA and histological grade. Therefore, any limitation in DRE-based cT assessment is not confined to T-staging nomenclature alone, but may also influence downstream risk stratification and treatment decisions, particularly because MRI-derived T-staging is not currently incorporated into any official risk stratification or prognostic tool [4]. Importantly, MRI-derived information is not included in either the UICC or AJCC TNM classification systems or AJCC prognostic stage grouping [5]. This MRI exclusion further underscores the significant disconnection with state-of-the-art clinical practice. While these international TNM-staging bodies formally mandate that imaging should not be used for clinical T-staging, in modern practice, we see that MRI has become indispensable in MDTs and patient counselling. MRI plays a critical role in local tumour classification, detailed localisation and anatomical staging, risk stratification, prognostication, and directing treatment. This modern practice creates a fundamental contradiction with the official TNM-classification guidelines.

## Arguments for MRI-based T-staging

### Flaws of digital rectal examination

DRE assesses only the posterior prostate surface and cannot reliably evaluate anterior, apical, basal, or seminal vesicle involvement, leading to both under- and overstaging [6]. In contrast, palpable abnormal prostate features on DRE suspected of having prostate cancer (e.g., stiff, hard nodularity, firmness, or irregular contour) may overlap with benign abnormalities, such as chronic inflammation or benign prostate hyperplasia. These false-positive findings may subsequently lead to wrongly higher risk profiles and unnecessary treatment escalation. Due to both false positives and negatives, the overall diagnostic accuracy of DRE is low and of very limited diagnostic value to prostate-specific antigen (PSA) testing [6, 7].

Despite the ease of using DRE, its value relies on the physician’s expertise, highlighting the test’s vulnerability [8]. DRE shows a low interobserver reproducibility, even among experts, emphasising the limitations of current DRE use in cT-staging [9]. While the UICC/AJCC bodies advocate that MRI should not be used for prostate cancer cT-staging, several of the arguments mentioned above can also be used to argue against using DRE. Therefore, an increasingly louder voice is calling for MRI to be used in clinical diagnosis and T-staging when available [6, 10–12].

### MRI outperforms DRE in diagnostic accuracy

MRI-based T-staging (mrT) informs key surgery decisions, including the feasibility of nerve-sparing surgery, bladder neck or urethral preservation, and the need for extended pelvic lymph node dissection during radical prostatectomy, with the help of other modalities like PSMA-PET-CT. In radiotherapy and active surveillance pathways, MRI findings may exclude patients from brachytherapy, guide the duration of adjuvant androgen-deprivation therapy (ADT), and identify those suitable for hypo-fractionated procedures with boost radiotherapy, thereby enabling treatment intensity to be matched to each individual’s risk profile [1, 13, 14].

In a prospective cohort, MRI demonstrated significantly higher diagnostic accuracy than DRE for preoperative T-staging (91.9%, 95% CI: 86.1–95.5% vs. 76.5%, 95% CI: 68.7–82.9%; *p* < 0.001), driven by substantially improved sensitivity (92.8% vs. 74.2%, *p* < 0.001) with comparable specificity (89.7% vs. 82.1%, *p* = 0.375) [12]. Multiple additional cohorts consistently demonstrate superior accuracy and sensitivity of MRI over DRE for the detection of ≥ T3 disease, confirming MRI’s robustness across different clinical settings [6, 12]. MRI remains the principal imaging modality for local anatomical T-staging because of its superior resolution and soft-tissue contrast for depiction of local tumour extent. PET-based techniques, particularly PSMA-PET, appear most relevant for nodal and distant staging. Future work should examine the added incremental value to local staging frameworks with MRI.

MRI has also shown superior performance compared with traditional DRE-based nomograms for predicting organ-confined disease. In a radical prostatectomy cohort, MRI achieved higher discriminative ability than the Partin tables (AUC 0.88 vs. 0.70), with further improvement after PSA adjustment (AUC 0.91). In one radical prostatectomy cohort, direct incorporation of MRI-derived T-stage into the Partin model did not improve predictive performance and, in that setting, was associated with lower discrimination. These findings suggest that MRI-derived T-stage may not be directly interchangeable with DRE-based variables within established legacy risk calculators. Rather, they support the view that established clinical parameters may be better used to complement and augment mrT-based assessment, instead of simply replacing it [15].

### MRI outperforms DRE in prognostication

MRI-derived staging categories (mrT3a and mrT3b) demonstrate stronger independent prognostic value than traditional clinical T-staging based on DRE (cT3a and cT3b) for both biochemical recurrence and metastatic failure [16, 17]. In contrast, clinical T3 disease identified by DRE represents a smaller, highly selected subgroup with adverse biological features in comparison with MRI-detected T3 disease, including older age, higher PSA, a greater proportion of Grade Group 4–5 cancers, a higher metastasis rate (16% vs. 5%, *p* < 0.001), and worse overall survival (HR 4.6 [95% CI: 1.4–15.0], *p* = 0.002) [11]. This reflects stage migration rather than conflicting prognostic signals. MRI-based T-staging remains a substantially stronger prognostic factor for biochemical recurrence (mrT3a: HR 2.16 [95% CI: 1.84–2.54]; mrT3b: HR 2.74 [2.06–3.65]) than DRE-based T-staging (cT3: HR 1.50 [1.25–1.80]), relative to T1-2 disease and adjusted for clinical covariates [18]. MRI-defined mrT3a and mrT3b were also independently associated with metastatic failure (HR 3.18 [2.04–4.97] and HR 5.58 [1.15–27.13], respectively).

### Increasing global MRI availability

Recently, there has been a significant global increase in MRI utilisation worldwide as we move towards MRI-guided diagnostic pathways [1, 18]. In the United States, pre-biopsy MRI use rose from 0.5% in 2007 to 35.5% in 2022 [19]. Similarly, another large cohort showed an increase from 1.5% in 2012 to 30.3% in 2021, reaching nearly 60% MRI utilisation among men with prior negative biopsies [20]. Beyond high-income regions, the *Lancet* Commission on Prostate Cancer (2024) highlighted an ongoing global transition towards earlier diagnosis, with MRI emerging as a scalable and cost-efficient technology that can help shift prostate cancer presentation from advanced to curable stages, even in low- and middle-income countries where late diagnosis remains the norm [21]. These converging trends, together with decreasing MRI costs, the emergence of zero Helium scanners with reduced energy consumption, and improvements in cost-effectiveness, have driven increased investment in MRI infrastructure, workforce training, and expertise worldwide, reflecting MRI’s growing central role in precision diagnosis and prostate cancer control.

Moreover, as increasing evidence demonstrates comparable diagnostic accuracy for MRI with and without dynamic contrast-enhanced imaging, MRI utilisation is used in both clinical pathways and population-level screening initiatives [22–24]. The omission of intravenous contrast medium injection not only simplifies workflow and reduces costs but also broadens accessibility by lowering technical and logistical barriers for healthcare systems [25].

In parallel, additional efficiency gains stem from shorter and task-specific MRI protocols, which maintain diagnostic performance while substantially reducing examination times, an important consideration given the anticipated surge in MRI demand [26]. Given that T2-weighted imaging is the cornerstone sequence for local staging, maintaining its quality is essential. Motion artefacts remain the primary source of degradation but can be mitigated through shorter acquisition protocols enabled by deep learning reconstructions (DLR), as well as through measures such as rectal preparation and the use of antiperistaltic agents. DLR can accelerate T2-weighted and diffusion-weighted imaging acquisitions by two to seven times while preserving, or even improving, image quality [27].

## MRI-based T-staging parallel to DRE-based T-staging

Clinicians worldwide already base key management decisions on MRI-derived tumour characteristics, as these metrics may more reliably stratify risk and better inform surgical and radiotherapy planning than DRE-based cT-staging alone. This structural misalignment between current T-staging and modern prostate cancer management argues for the inclusion of MRI in the official TNM classification system, or at least for its parallel reporting as a distinct category alongside clinical T-staging (Table 1b). In practice, parallel reporting means that the conventional TNM clinical T-stage should continue to be assigned according to current guideline definitions based on DRE, while MRI findings should be reported separately as an imaging-based local staging assessment (mrT). In context, a patient may be assessed as cT2-mrT3a when DRE suggests organ-confined disease and MRI shows imaging signs of extraprostatic extension. This discordance may reflect MRI detection of subtle local extension not captured by DRE. A cT3-mrT2 discordance reflects the limitations of either palpation specificity or MRI sensitivity, depending on the clinical context. This approach preserves compatibility with existing guideline-based risk stratification while allowing prospective evaluation of staging discordance, treatment changes, and outcome implications. Discordant cT and mrT findings should not automatically be interpreted as error; rather, they should be recognised as biologically and clinically informative differences between palpation-based and imaging-based assessment. and should therefore be considered for multidisciplinary review and discussion. This approach preserves compatibility with existing guideline-based risk stratification while enabling prospective study of staging discordance, treatment modifications, and outcome implications. Although DRE-based cT-staging may progressively diminish in importance as MRI becomes clinically validated and society-endorsed for formal staging use, this evolution will likely be gradual because TNM must remain applicable in settings where MRI is not universally available. In that respect, parallel cT and mrT reporting may serve as a pragmatic transitional framework before any future formal integration of MRI into staging systems.

### MRI challenges

Although MRI improves the accuracy of T-staging, several critical issues hamper its widespread adoption and formal incorporation into the official UICC/AJCC editions of the TNM-staging classification for prostate cancer. First, prostate MRI still shows significant variation in accessibility, image quality, and radiologist expertise across institutions, resulting in inconsistent T-staging outputs [28]. Second, real-world data demonstrate that MRI-based T-staging remains limited in diagnostic accuracy compared to the gold standard of pathology. MRI can produce both false-negative and false-positive assessments when distinguishing organ-confined (T2) from extraprostatic (T3) disease [28, 29]. In the evaluation of extraprostatic extension (EPE) on prostate MRI, reinterpretation by subspecialised genitourinary oncologic radiologists significantly improved sensitivity and overall diagnostic performance compared with initial readings, highlighting the importance of reader expertise in accurately identifying EPE [30]. In a multi-institutional study including 6253 patients, MRI exhibited high specificity (93–99%) but low sensitivity (17–37%) for key MRI-staging parameters such as EPE, seminal vesicle invasion (SVI), and bladder-neck invasion [31]. The low sensitivity of MRI in T-staging reinforces pathology as the ultimate gold standard, and yet not a reliable substitute. However, in contrast to radical prostatectomy treatment, pathological T-staging is not available in radiation or focal treatment regimen. Therefore, MRI should be viewed as complementary to pathology, rather than a competing modality. Physicians increasingly make MRI-based clinical decisions before pathological confirmation, or other key prognostic variables are not yet known but may substantially influence management decisions [32].

It has long been recognised that MRI-based T-staging performance is risk-dependent. In low-risk disease, radiologists tend to maximise the negative predictive value, favouring T2 assignment, whereas in high-risk disease, interpretation shifts toward maximising the positive predictive value, having a lower threshold for calling T3a disease. This risk-adapted behaviour is supported by the increasing prevalence of EPE across risk categories, reported as approximately 20% in low-risk, 58% in intermediate-risk, and 77% in high-risk patients [33]. In any case, the challenge of consistently interpreting subtle imaging features of extraprostatic extension leads to low interobserver reproducibility in MRI-based T-staging [34].

### Stage shift and risk migration

**Fig. 1:**
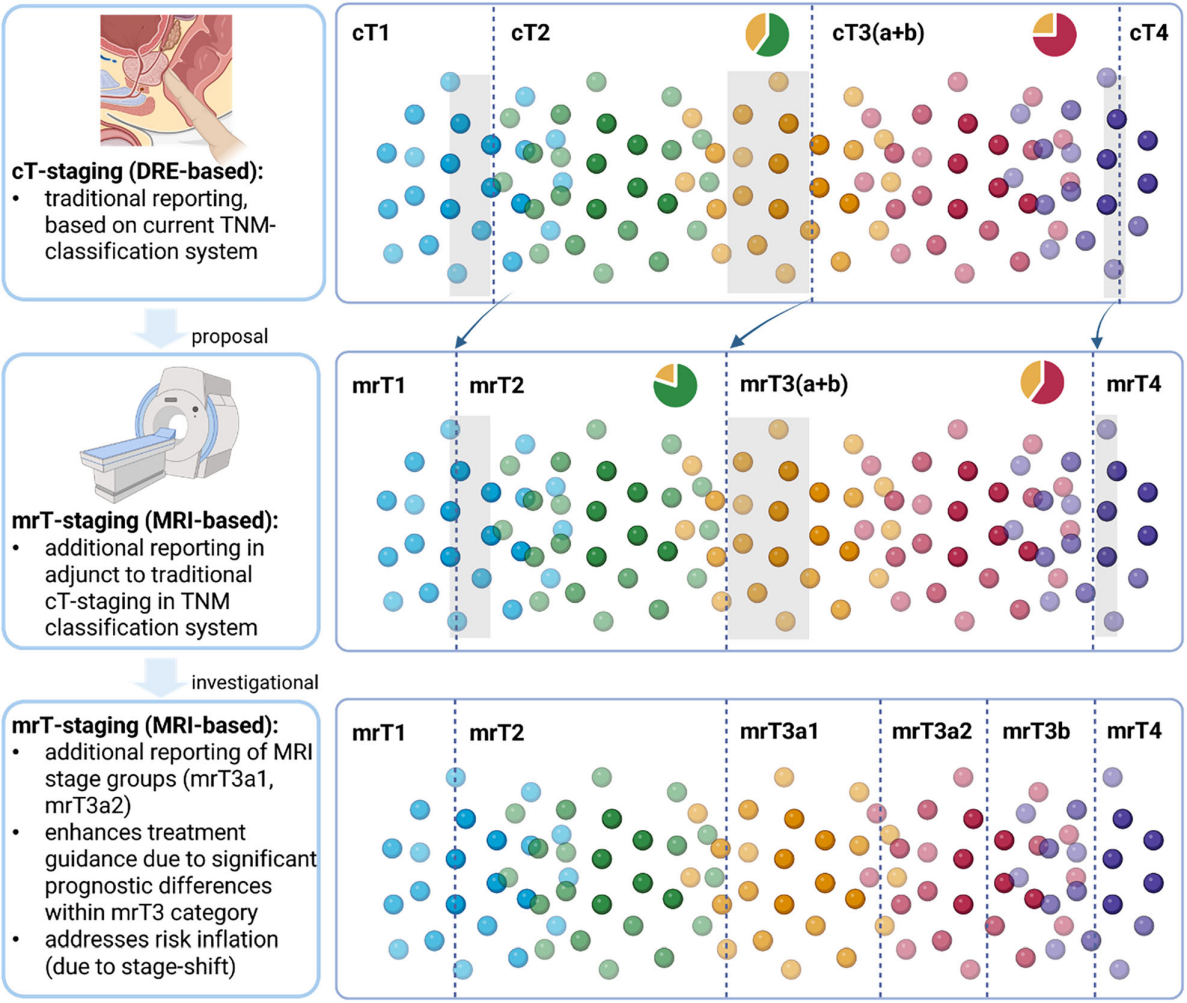
Illustration of stage migration when transitioning from DRE-based clinical T-staging (cT) to MRI-based T-staging (mrT). Because MRI is more sensitive than DRE for detecting subtle or minimal extraprostatic extension, a proportion of men originally classified as cT2 are reclassified as mrT3. This reallocation, shown by the shift of the dotted T-stage boundaries to the left (middle row), results in stage migration (light grey box), whereby biologically more favourable tumours move into higher MRI-defined stages. Consequently, outcomes appear better for mrT2 and mrT3 groups than for their cT equivalents, not necessarily because prognosis has improved, but because of the inclusion of lower-risk disease. The colour transition of bubbles (from blue to purple) illustrates the full disease spectrum from low- to very-high-risk prostate cancer and highlights how risk distribution changes across staging systems. MRI further enables separation not only between mrT2 and mrT3, but also within the T3 category, distinguishing focal from established extraprostatic extension (mrT3a1, mrT3a2) and seminal vesicle invasion (mrT3b). This granularity surpasses the traditional dichotomous clinical system (cT2 vs. cT3), capturing the continuum from organ-confined disease to advanced local extension. Together, these refined MRI-based subgroups provide a conceptual framework for an updated, imaging-driven T-staging system in prostate cancer. This figure was created in BioRender. Agrotis, G. (2026) https://BioRender.com/hn6zr6q

Beyond the challenges directly related to MRI-based T-staging, the introduction of a sensitive staging test inherently induces risk inflation owing to a stage shift [16]. This can profoundly impact prognosis and treatment management. MRI identifies subtle or minimal EPE (mrT3a) that would remain clinically unapparent on DRE (cT2). This upstaging from cT2 to mrT3a creates a stage shift toward locally advanced disease, artificially improving the prognosis of the newly radiologically (mrT) and clinically staged (cT) cohorts (Fig. 1). Consequently, radiologically T-staged patients may appear to have better long-term survival outcomes, while the pathological prostate cancer stage of these patients has not changed [16, 35]. Importantly, MRI-driven upstaging should not be interpreted as proof of worse intrinsic tumour biology in all cases. Rather, improved local staging may redistribute patients across stage categories and alter the apparent prognosis of both lower- and higher-stage groups without changing the underlying pathological disease burden. This distinction is critical when interpreting differences in survival between DRE-based and MRI-based stage groups.

Traditional EAU, NCCN, and D’Amico risk groups were all validated using DRE-based staging. Simply substituting DRE-based T-staging with MRI-based T-staging, therefore, introduces risk migration and may distort the calibration of historical prognostic models. To quantify and appropriately adjust for this transition, both MRI-based and DRE-based T-staging should be reported in parallel. Furthermore, upstaging from DRE-based cT2 to MRI-based mrT3a due to minimal EPE may lead to treatment escalation, for example, short- or long-term androgen-deprivation therapy (ADT), even when the biological implications of such subtle disease remain uncertain. Prospective datasets should therefore capture both cT and mrT classifications together with downstream treatment decisions and long-term outcomes.

### Adjustments to risk migration—introducing MRI-based prognostic groups

To account for anticipated risk migration, new risk groups calibrated to MRI-based T-staging must be developed. These new risk groups should integrate both radiological and clinical parameters to redefine the risk of having “organ-confined” or “locally advanced” disease. These risk groups need to be validated, along with pathology standards and long-term oncological outcomes. In comparison with the standard DRE-based model, the modified EAU risk classification incorporated MRI-based T-stages (T1/T2ab, T2c, T3, T4) to better predict progression-free survival [16]. Furthermore, the extent of EPE distinguished by focal and established disease (i.e., mrT3a_1_ and mrT3a_2_; “Focal and established extraprostatic extension within MRI-based T-staging” section) could also be incorporated into a newly MRI-calibrated system. Such MRI-calibrated systems would preserve prognostic accuracy, minimise stage migration, and ultimately allow MRI to serve as the reference for local T-staging in the TNM classification, reflecting better the true extent of disease.

## Focal and established extraprostatic extension within MRI-based T-staging

In MRI, assessment of extraprostatic extension (EPE) is currently based on the combination of direct and indirect imaging features rather than on any single sign. Common suspicious findings include broad prostatic boundaries contact, bulging or irregularity, loss of the rectoprostatic angle, and measurable tumour extension into the periprostatic fat. Because individual features vary in sensitivity, specificity, and inter-reader agreement, MRI-based scoring systems were developed to support a more structured staging assessment by integrating multiple findings rather than the interpretation of isolated features. This structured approach provides the biological and radiological basis for distinguishing organ-confined disease from focal or established EPE in MRI-calibrated staging systems. However, it remains unclear whether individual MRI features themselves carry independent prognostic significance, or whether binary classification systems based on the overall presence or absence of EPE are more clinically relevant [29].

Although overall EPE is traditionally considered an adverse pathological feature, several long-term series suggest that T3a disease has a limited impact on long-term outcomes in the modern treatment era [36, 37]. Similarly, histopathological studies have shown that increasing radial or circumferential length of EPE, rather than its mere presence, drives the excess risk of recurrence, with very small foci behaving closer to organ-confined tumours after adjustment for grade and margin status [38–40]. However, the distinction between “focal” and “established” EPE remains incompletely standardised, even in pathology. Proposed pathological definitions have ranged from descriptive criteria such as a few glands beyond the prostate or involvement on one to two sections, to quantitative radial cut-offs such as 0.5 mm, 0.75 mm, or 0.8 mm. This lack of consensus is important because such microscopic definitions do not translate directly to MRI, which cannot resolve individual glands and is constrained by its spatial resolution (new ref). Accordingly, any MRI-based distinction between focal and established EPE should be regarded as an image-based construct, informed by what MRI can realistically depict, rather than as a direct surrogate for histopathological microinvasion. Radiological focal EPE refers to limited or subtle MRI evidence of tumour extension beyond the prostatic borders, such as minimal breach or suspicious boundary irregularity, without gross, measurable extraprostatic soft-tissue extension. Established EPE refers to clear measurable tumour tissue extending beyond the prostate into periprostatic fat.

From a prognostic and therapeutic standpoint, this limitation may not be critical. MRI’s reduced sensitivity for microscopic or minimal EPE may be acceptable if the clinically relevant target is the identification of established EPE, which is more likely to influence recurrence risk, surgical margin status, and treatment planning [41].

**Fig. 2:**
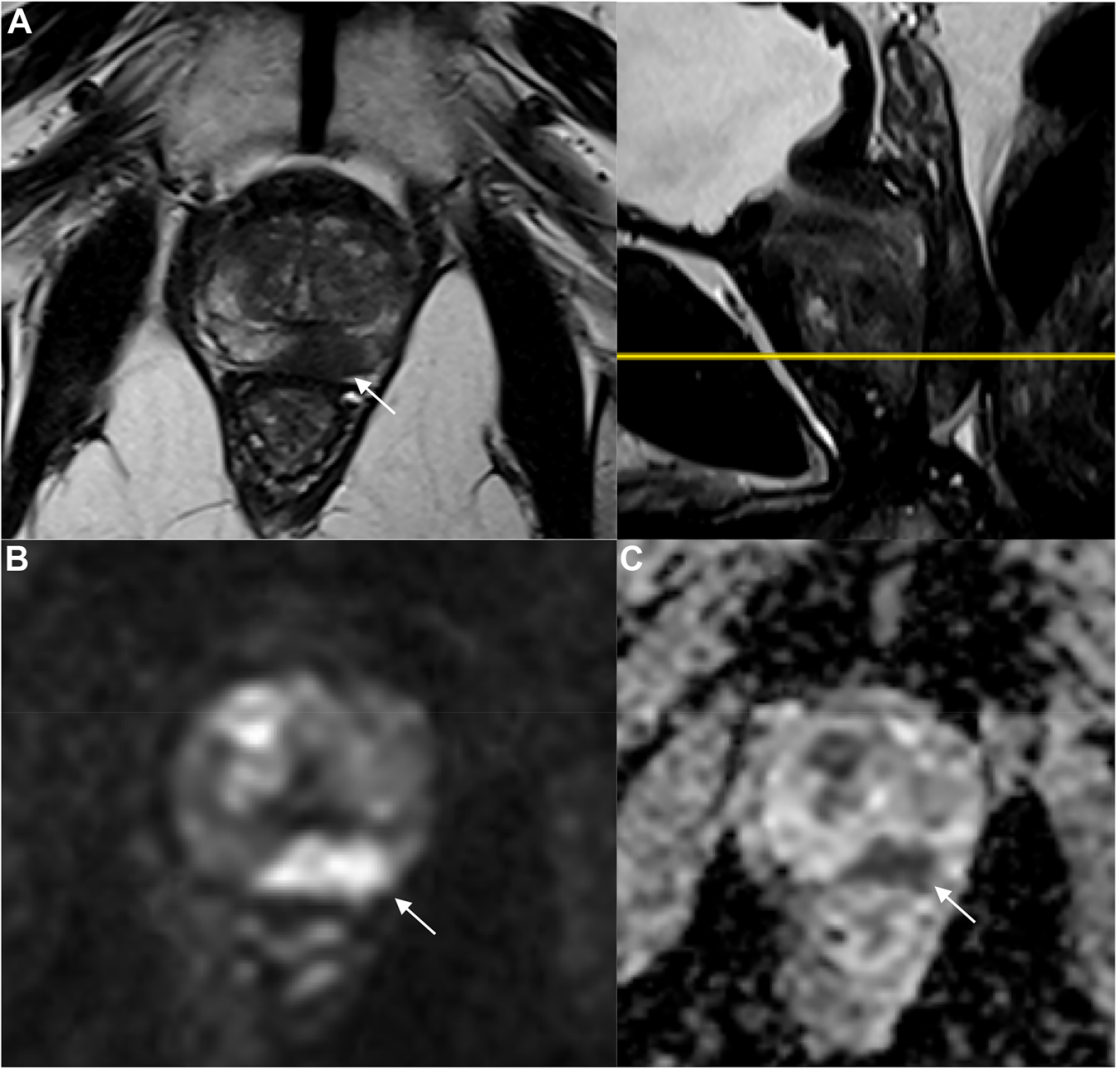
Posterolateral mid-apical lesion with focal extraprostatic extension. Multiparametric MRI demonstrates a posterolateral lesion at the mid-apical level, measuring 16 mm in maximal axial diameter. On axial T2-weighted imaging (**A**), the lesion shows low signal intensity with subtle spiculation and irregularity of the borders, without definite breach or measurable extraprostatic tumour extension. The total capsular contact length measures 18 mm. Diffusion-weighted imaging and ADC mapping (**B**, **C**) demonstrate corresponding diffusion restriction. These imaging findings are consistent with minor MRI features suggestive of extraprostatic extension. Histopathological analysis following radical prostatectomy confirmed ISUP Grade Group 4 (Gleason score 4 + 4 = 8) with focal extraprostatic extension (pT3a)

**Fig. 3:**
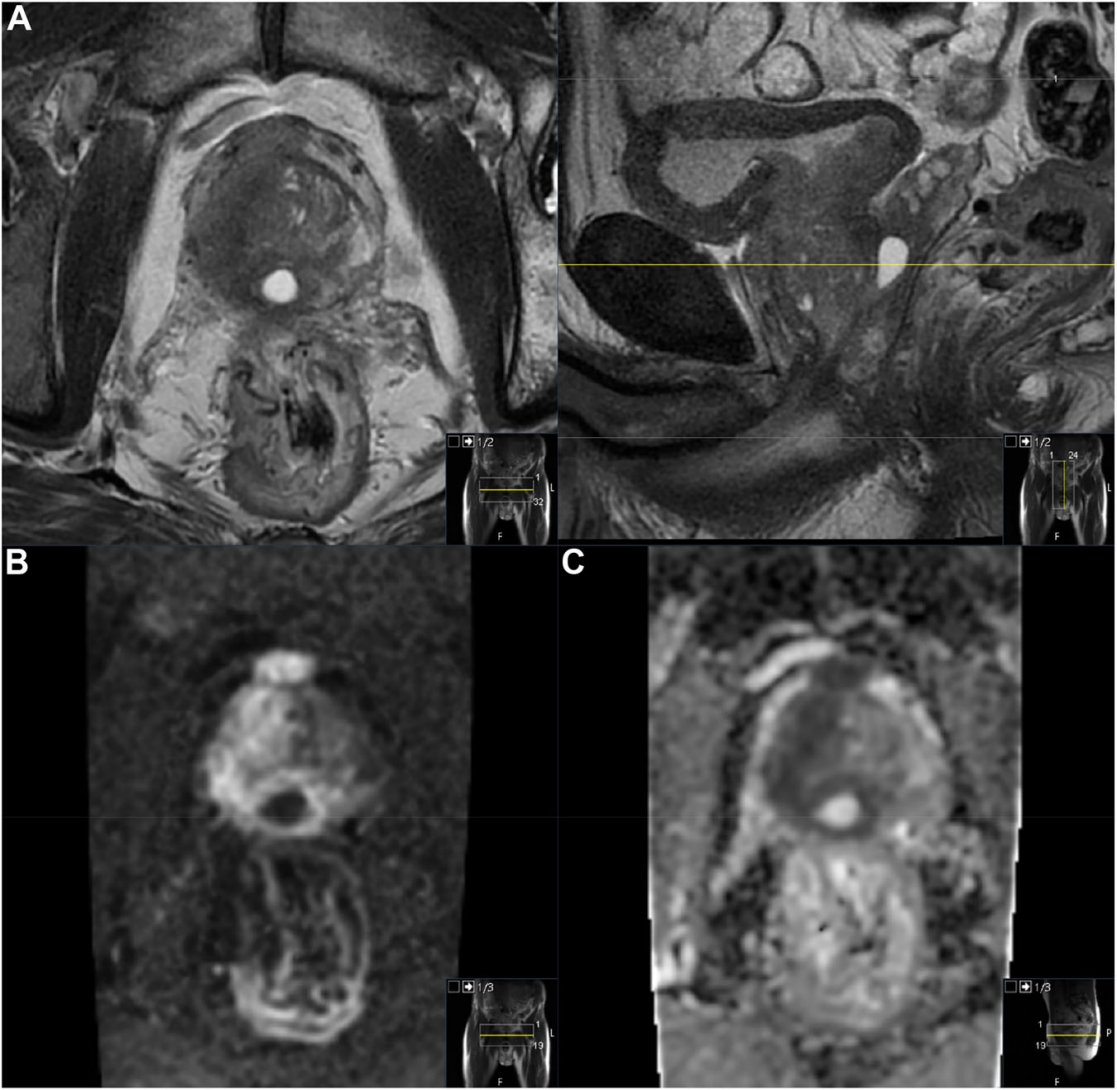
Anterior prostate cancer with established extraprostatic extension (mrT3a_2_). Multiparametric MRI demonstrates an anterior mid-gland lesion with measurable extraprostatic extension. On axial T2-weighted imaging (**A**), the lesion shows low signal intensity with disruption of the anterior fibromuscular stroma and extension into the periprostatic fat, measuring 9 mm beyond the borders at the mid-gland level. Diffusion-weighted imaging (b = 1400 s/mm²) (**B**) and corresponding ADC map (**C**) show marked diffusion restriction, consistent with high-grade disease. Histopathological correlation following radical prostatectomy confirmed ISUP Grade Group 5 (Gleason score 4 + 5 = 9) with established extraprostatic extension (pT3a)

**Fig. 4:**
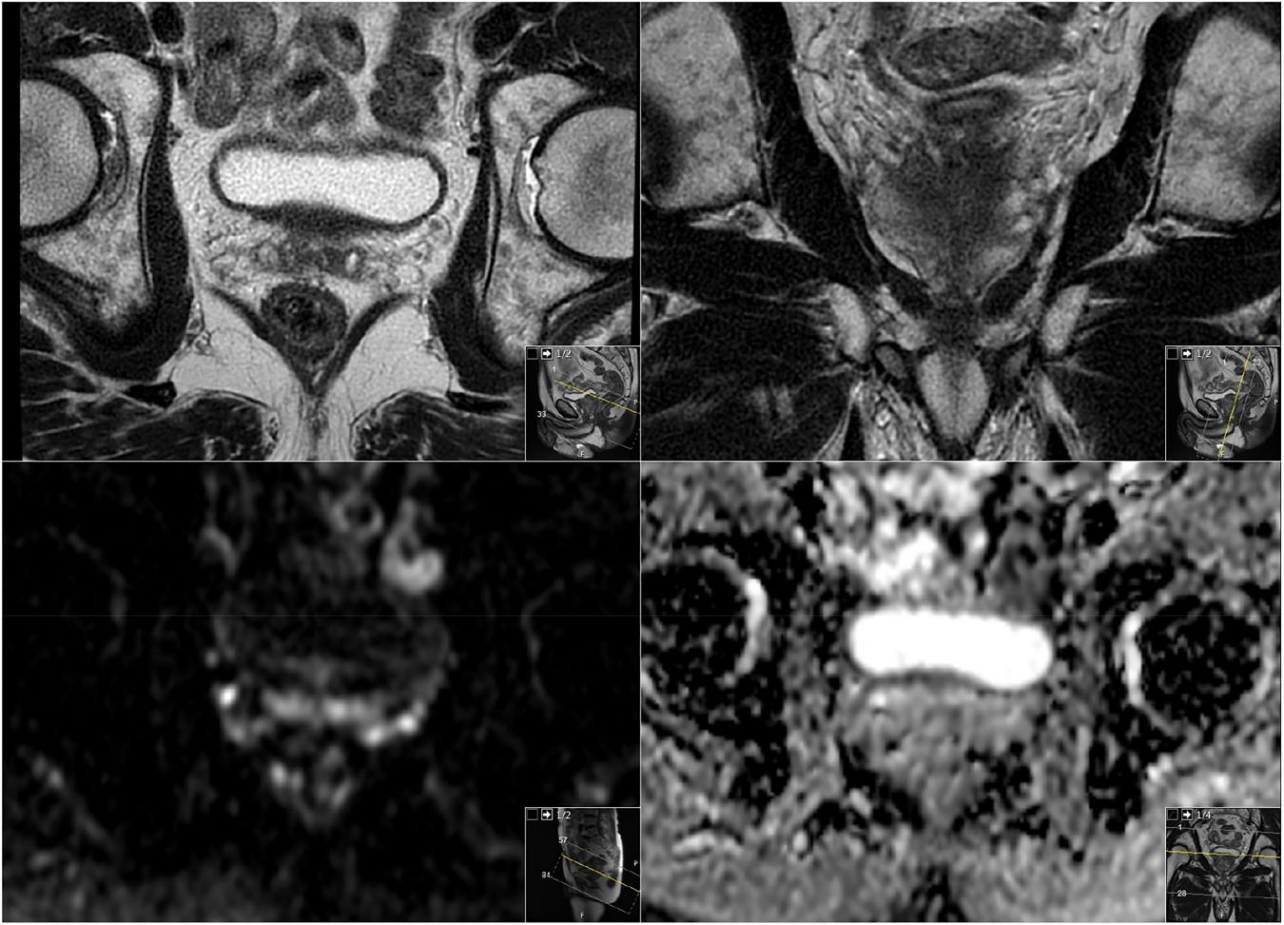
Minor seminal vesicle involvement (mrT3b_1_). At the left posterolateral aspect of the prostate base, there is a subtle asymmetric low T2 signal intensity and focal diffusion restriction extending into the proximal left seminal vesicle. The findings are consistent with minor seminal vesicle invasion, representing early contiguous spread from the primary tumour. Histopathology confirmed limited seminal vesicle involvement, corresponding to pT3b disease

**Fig. 5:**
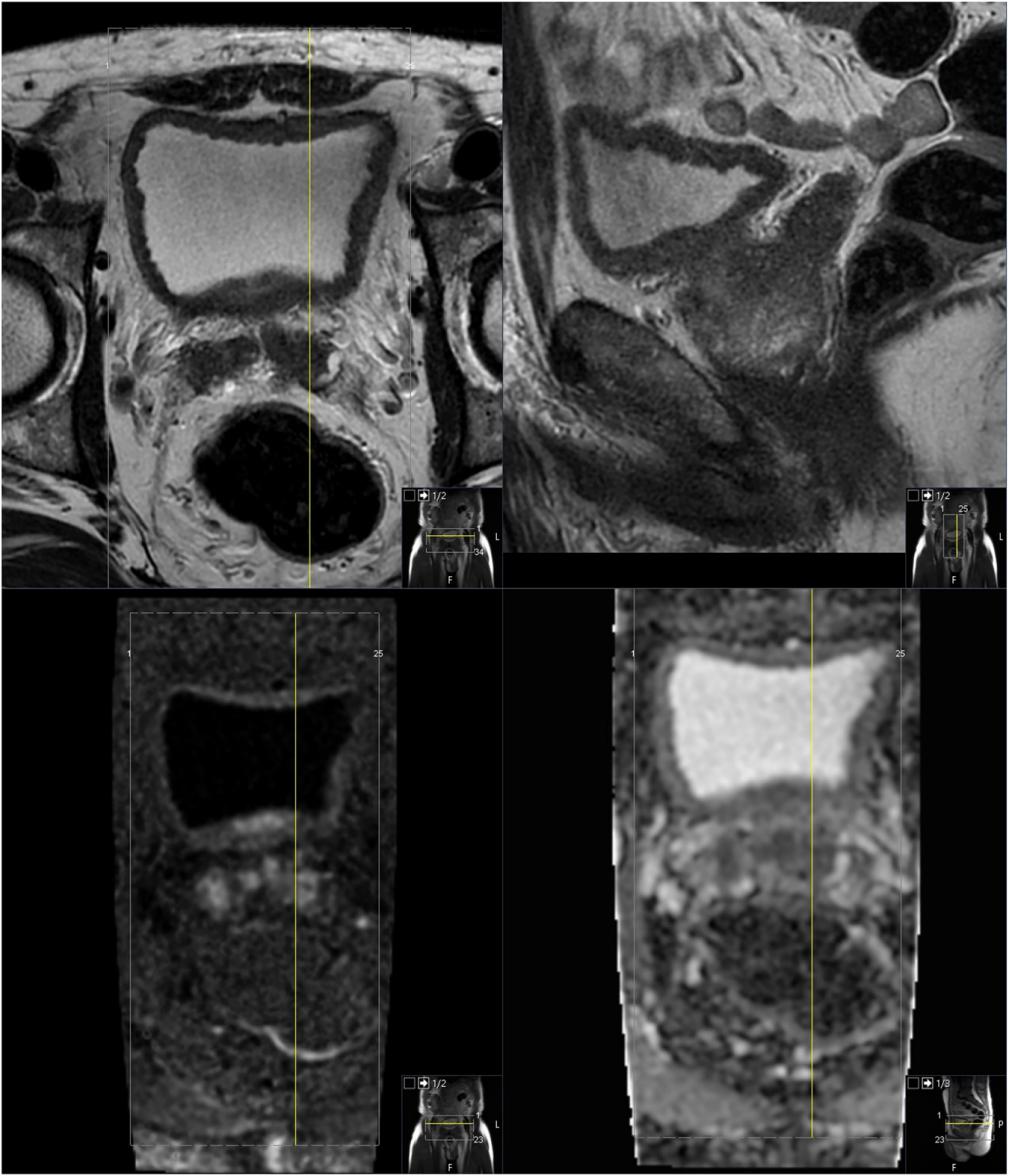
Major bilateral seminal vesicle invasion (mrT3b_2_). MRI demonstrates extensive bilateral invasion of the seminal vesicles, characterised by low T2 signal intensity with loss of normal vesicular architecture, marked diffusion restriction, and contiguous tumour extension from the prostate base into the bodies of both seminal vesicles, replacing the normal high T2 intraluminal fluid signal. Histopathological correlation following radical prostatectomy confirmed bilateral seminal vesicle invasion, corresponding to pT3b disease

Consequently, MRI offers the opportunity to stratify disease by its radiological resolution capabilities into focal versus established EPE, categories that carry distinct prognostic implications, which may help resolve stage migration concerns (Fig. 1) [42]. Identification of minimal or subtle prostatic border breach (focal EPE, mrT3a1) (Fig. 2) versus gross extension beyond the borders of the prostate (established EPE, mrT3a2) (Fig. 3) can inform nerve-sparing surgical planning and duration of adjuvant therapy decisions, aligning imaging interpretation with histopathological definitions. However, radiological evidence directly correlating MRI-defined focal EPE with established EPE and long-term prognostic outcomes is lacking. Future work must therefore focus on correlating radiological focal and established EPE parameters with postoperative outcomes, margin status, and recurrence risk to confirm their prognostic value and justify their incorporation into preoperative treatment decision-making algorithms. This also accounts for SVI with limited or focal disease (mrT3b_1_) (Fig. 4) and more established disease (mrT3b_2_) (Fig. 5). Radiological focal EPE refers to limited or subtle MRI evidence of tumour extension beyond the prostatic borders, such as minimal breach or suspicious irregularity, without gross measurable extraprostatic extension. Established EPE refers to clear measurable tumour tissue extending beyond the prostate into periprostatic fat. MRI-based staging should be viewed not merely as complementary to DRE, but as a more objective and potentially more reproducible method for assessing local tumour extent. This is particularly relevant because, in contemporary practice, some centres have already adopted MRI-first or “straight-to-test” pathways in which prostate MRI is performed and reported before the first urological consultation, reducing the practical role of DRE in initial staging assessment. Just as imaging has replaced predominantly clinical assessment in other organ systems, prostate cancer T-staging is likely to evolve toward an MRI-based framework for prostate local tumour extent (Fig. 1). Therefore, incorporating MRI-based T-staging as an independent component within or alongside the TNM classification system will modernise prostate cancer T-staging, improve risk stratification, and facilitate treatment individualisation based on objective radiologic evidence rather than subjective clinical palpation.

## MRI-based N and M staging

While MRI provides the most established imaging approach for local staging of the primary tumour (T), its performance in nodal staging (N) is limited, with low sensitivity compared to the invasive yet highly accurate extended pelvic lymph node dissection [43, 44]. This limitation is primarily due to reliance on morphology and size-based criteria and shows low sensitivity but high specificity, failing to detect micro-metastases [45]. Nanoparticle-enhanced MRI, particularly using ultrasmall superparamagnetic iron oxide (USPIO) agents, has demonstrated promise in improving N-staging in prostate cancer, achieving high sensitivity and specificity for detecting nodal metastases [46, 47]. Despite these strong diagnostic results, clinical implementation remains restricted by regulatory challenges and the limited availability of contrast agents.

Whole-body MRI (WB-MRI) demonstrates high diagnostic performance for detecting and monitoring metastatic (M) bone disease and soft-tissue deposits, with superior sensitivity and specificity for bone metastases compared with bone scintigraphy (BS) and computed tomography (CT) [48]. In a single-step approach, WB-MRI can stage the tumour (T) and identify nodal (N) and distant metastases (M). It helps clarify potential false-positive bone lesions observed on Prostate-Specific Membrane Antigen Positron Emission Tomography (PSMA-PET). However, it may need to be complemented by chest CT for a comprehensive lung assessment [49]. WB-MRI achieves higher sensitivity than BS-CT (98–100% vs. 86%) for M-staging and comparable sensitivity to CT (77–82%) for N-staging [50]. Comparative studies show similar performance between WB-MRI and PSMA-PET for detecting distant metastases, though WB-MRI is slightly less effective for nodal disease. Importantly, in men with metastatic hormone-sensitive prostate cancer, WB-MRI detected more bone-only metastases and predicted overall survival more accurately than BS-CT [51].

## Reporting MRI-based T-staging

### mrT1 cancer (MRI-occult disease)

Clinical staging (cT) remains rooted in DRE, which relies on palpability rather than anatomical visualisation. Consequently, cT1 tumours are defined as non-palpable lesions detected incidentally by biopsy, and there is no pathological equivalent (no pT1 category). Similarly, mrT1 tumours can be defined when lesions are classified as MRI-occult or non-visible (Table 1). MRI-based T-staging, therefore, provides a fundamentally different dimension of assessment. Rather than evaluating palpability, it assesses the visibility of anatomical extent and local invasion, based on high-resolution soft-tissue contrast.

### mrT2 cancer (organ-confined, MRI-visible disease)

mrT2 disease refers to MRI-visible prostate cancer confined within the prostatic borders. In addition to cT2 labelling any palpable tumour as organ-confined, mrT2 staging reflects true anatomic confinement, relying on clear delineation of the lesion, intact capsule-tumour interface, and absence of imaging signs of EPE. MRI therefore provides a more accurate representation of organ-confined disease, integrating tumour volume, zonal anatomy, and tumour-capsule contact length to refine prognostic interpretation beyond the binary cT2 classification.

### mrT3 cancer (extraprostatic extension and seminal vesicle invasion)

Unlike cT3, which is based solely on palpable fixation, MRI visualises the prostate borders, periprostatic fat, and seminal vesicles, enabling a more nuanced T-classification that also carries prognostic significance. mrT3a disease is defined as EPE without SVI. Current radiological classification does not distinguish between focal and established EPE, whereas pathological classification does. mrT3b is assigned when the tumour demonstrates true seminal vesicle invasion, either extending directly beyond the prostatic base into the seminal vesicle muscle tissue or with other infiltrative pathways. This represents a biologically more aggressive stage associated with markedly worse oncologic outcomes [36].

### mrT4 cancer (invasion of adjacent structures)

**Fig. 6:**
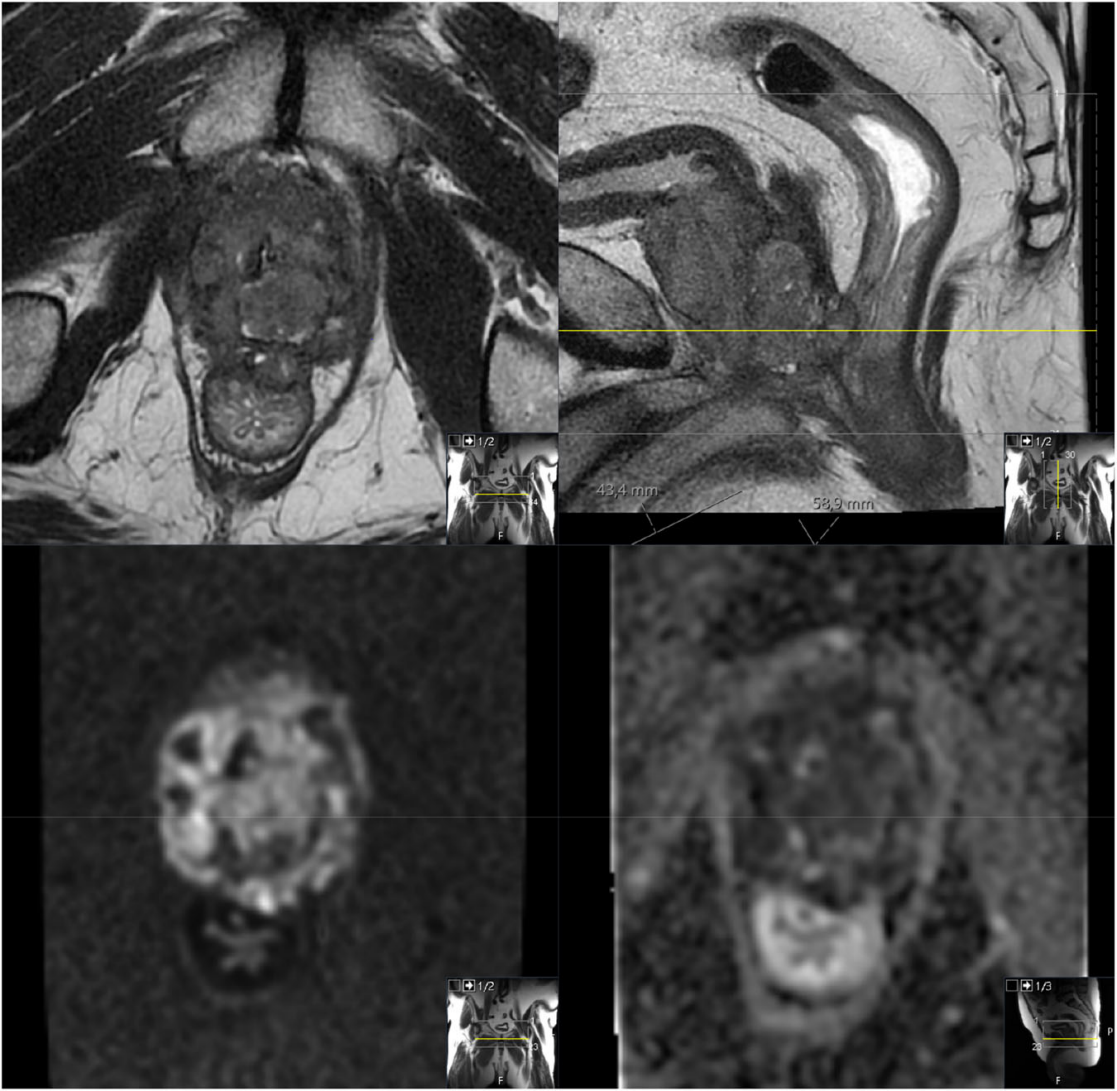
Locally advanced prostate cancer with rectal wall invasion (T4). MRI demonstrates a large infiltrative posterior prostate lesion with low T2 signal intensity and ill-defined margins, extending beyond the posterior prostatic borders into the anterior rectal wall. There is obliteration of the rectoprostatic fat plane, with direct tumour contact and infiltration of the rectal muscularis propria, associated with marked diffusion restriction. These findings are consistent with direct invasion of adjacent organs (T4 disease). Patient underwent radiotherapy and long-term hormonal therapy

mrT4 staging reflects invasion of structures beyond the periprostatic fat and seminal vesicles, including the bladder, external sphincter, rectum, pelvic floor musculature, or pelvic sidewall (Fig. 4). These findings signify locally advanced disease with very-high-risk biology, limited suitability for surgical intervention, and the near-universal need for multimodality therapy. MRI is critical for detecting these advanced patterns of invasion, which are not assessable through DRE (Fig. 6).

## Towards a universal MRI-based T-staging system

Historically, MRI-based scoring systems have prioritised sensitivity over specificity to avoid undertreatment, emphasising even the detection of subtle or equivocal findings. Yet in prostate cancer, a sensitivity-driven paradigm may reduce specificity and lead to overstaging and overtreatment. Conversely, insufficient sensitivity for established extraprostatic extension or seminal vesicle invasion may contribute to undertreatment through under-recognition of clinically meaningful local spread [29, 52]. Modern cancer care should shift from “detecting more” towards “treating smarter”, underscoring the requirement for imaging criteria that are reproducible, clinically meaningful, and strongly linked to patient outcomes (Table 2). MRI-based T-staging plays a central role in contemporary prostate cancer management, providing superior diagnostic and prognostic information compared with clinical DRE-based T-staging. MRI-based T-staging generally outperforms DRE T-staging in delineating tumour extent, improving risk stratification, and guiding treatment decisions. Therefore, integrating MRI into future revisions of the TNM classification system, through parallel reporting alongside clinical T-staging, represents an important step toward resolving the structural misalignment between modern imaging practice and official prostate cancer staging frameworks. As MRI becomes more accessible and affordable, cost-efficient pathways will facilitate broader adoption across less-resourced healthcare settings.

**Table 2:** Perspectives on MRI-based T-staging

Domain	Perspectives
Clinical T-staging	Digital rectal examination (DRE) alone is insufficient for accurate local T-staging in contemporary prostate cancer care.
Role of MRI	MRI provides superior anatomical and prognostic assessment of local tumour extent compared with DRE and should be used for local staging when available.
Parallel reporting	MRI-based T-staging (mrT) and DRE-based clinical T-staging (cT) should be reported in parallel rather than used interchangeably.
Stage migration	MRI-based T-staging introduces stage shift and risk migration, which must be explicitly acknowledged in clinical interpretation and research.
Prognostic value	MRI-based T-staging demonstrates stronger independent prognostic value for oncologic outcomes than DRE-based T-staging.
EPE and SVI stratification	MRI-based T-staging should differentiate focal from established disease in extraprostatic extension and seminal vesicle invasion to better reflect biological risk.
Clinical decision-making	MRI-based T-staging should guide pre-treatment decision-making, including nerve-sparing surgery, radiotherapy planning, and systemic therapy intensification.
Quality assurance, Reader expertise	Accuracy of MRI-based T-staging is dependent on image quality and reader expertise; subspecialty training is essential.
Reference standard	Pathology remains the definitive staging reference; MRI should be viewed as a complementary pre-treatment staging tool.
TNM revision	Formal integration of MRI into TNM classification requires prognostic grouping also based on MRI tumour characteristics, validated against pathology and long-term oncological outcomes.

For such a system to be broadly applicable and clinically credible, several key prerequisites must be met: (1) precisely defined imaging criteria that demonstrate strong inter-reader agreement; (2) systematic validation against robust reference standards, including whole-mount histopathology when available; (3) technical adaptability, ensuring reliability across MRI platforms, field strengths, and institutional protocols; and (4) prognostic relevance, with clear correlations to outcomes such as positive surgical margins, biochemical recurrence, and long-term prostate cancer specific survival [53]. This strategy allows for a staging system that minimises overcalling, thereby aligning MRI interpretation with surgical planning, prognostic outcomes, and patient-centred care. To translate MRI-based T-staging from parallel reporting into a formally adopted staging framework, a structured and evidence-driven research roadmap is required (Table 3).

**Table 3:** Roadmap towards a consensus MRI-based T-staging system

Steps	Aim	Key activities
1	Establish prognostic relevance of MRI-based T-staging	Demonstrate the independent prognostic value of mrT categories for long-term oncologic outcomes
2	Consolidate existing evidence	Updated systematic synthesis of MRI-based T-staging diagnostic performance for EPE and SVI
3	Evaluate staging discordance and migration	Head-to-head comparison of cT vs. mrT vs. pT for T2, T3a, T3b
4	Define MRI-based T-staging criteria that correlate with long-term outcomes	Develop reproducible MRI criteria (mrT1–mrT4); radiological focal vs. established EPE/SVI that correlate with pathology and long-term outcomes.
5	Achieve expert consensus	Structured Delphi process and consensus meeting
6	Develop MRI-calibrated prognostic groups	Integrate mrT with PSA, ISUP grade, and clinical risk factors
7	Validate across centres	Retrospective and prospective multicentre validation
8	International dissemination and implementation	Alignment with TNM international frameworks

## Summary and conclusion

MRI-based T-staging plays a central role in contemporary prostate cancer management, providing superior diagnostic and prognostic information compared with clinical DRE-based T-staging. Therefore, integrating MRI into future revisions of the TNM classification system through parallel reporting alongside clinical T-staging represents an important step toward resolving the structural misalignment between modern imaging practice and official prostate cancer staging. As MRI becomes more accessible and affordable, cost-efficient pathways will facilitate broader adoption in less-resourced healthcare settings.
